# Membranous Aplasia Cutis Congenita: A Case Report

**DOI:** 10.7759/cureus.117650

**Published:** 2026-10-08

**Authors:** Kiley B Kirby, Stephen Madaras, Matthew Willett

**Affiliations:** 1 Family Medicine, Naval Hospital Camp Pendleton, Oceanside, USA; 2 Dermatology, Naval Hospital Camp Pendleton, Oceanside, USA

**Keywords:** acc, aplasia cutis congenita, frieden classification, group 1 aplasia cutis congenita, membranous aplasia cutis congenita

## Abstract

Aplasia cutis congenita (ACC) is a rare congenital skin defect in neonates, most commonly located on the scalp. ACC phenotypes can range from ulcerated to scarred and vary in their underlying etiology, with many having associations with perinatal or obstetrical conditions. This case specifically describes the membranous subtype without multiple abnormalities. Most group 1 lesions heal over several months; in this case, no treatment was required, and the infant has continued to meet all developmental milestones through his first year of life.

## Introduction

Aplasia cutis congenita (ACC) is a rare congenital skin defect in neonates, with an estimated prevalence of approximately 0.5-3 cases per 10,000 live births [1,2]. Lesions are most commonly located on the scalp, accounting for 85% of solitary lesions [3]. Various stages of cicatrization can be present at birth, ranging from shallow erosions and open wounds to partially granulated or fibrosed tissue resulting from in utero healing [3]. In 1986, Frieden first proposed a set of nine clinical subtypes based on mode of inheritance, location, physical presentation, and associated malformations [4].

The nine subtypes vary in their underlying etiologies, but many have associations with perinatal or obstetrical conditions such as intrauterine infection, placental infarction or other ischemic events, intrauterine friction, obstetrical trauma, teratogenic medication, or any intrauterine event that disrupts skin development [3-7].

This case describes an infant with a lesion classified as Frieden group 1 ACC as follows: scalp ACC without multiple abnormalities, specifically the membranous subtype. The membranous variation is usually caused by sporadic mutations, whereas other localized scalp lesions that present as irregular, scar-like lesions are often inherited via autosomal dominant BMS1 gene mutations [3]. This subtype may also represent a partial manifestation of neural tube defects due to loss-of-function mutations of KCTD1/KCTD15 in neural crest cells, which can lead to incomplete formation of the cranial sutures and disruption of the overlying tissue [8,9]. Membranous ACC often presents with a ring of hypertrichosis surrounding an alopecic lesion, known as the "hair collar sign," which is associated with other neural tube defects such as meningoceles [8].

Histopathology, although not required for diagnosis, may demonstrate an extremely thin or absent epidermis and near-complete or complete loss of appendage structures [3,10,11]. These findings support a relationship to neural crest cells, as skin appendage morphogenesis is regulated by KCTD1/KCTD15 [9].

## Case presentation

A six-week-old Hispanic male infant born at 39 weeks 5 days of gestation to a 38-year-old mother was seen in clinic for evaluation of a scalp lesion that had been present since birth. The lesion initially appeared as a fluid-filled blister and spontaneously involuted over the following weeks. The mother’s prenatal course was complicated by advanced maternal age and a history of herpes simplex virus (HSV), for which valacyclovir was initiated at 36 weeks of gestation. There was no evidence of internal or external lesions upon admission to labor and delivery. The mother was Group B Streptococcus (GBS) negative, and the duration of membrane rupture was 1 h 58 min; there were no maternal fevers or additional infectious risk factors. The intrapartum course was significant for placement of a fetal scalp electrode (FSE) 2 h before delivery.

The infant was born with Apgar scores of 8 and 9, with points deducted only for color [12]. No significant trauma to the scalp was noted on initial neonatal examination, and the infant was discharged at approximately 24 h of life following a routine stay. At six weeks, examination revealed a well-developed infant meeting all expected developmental milestones. Cutaneous findings were significant for a 1 cm, well-circumscribed, flesh-colored alopecic nodule on the right side of the vertex scalp lateral to the midline surrounded by a ring of coarse, dark hair (Figure 1). Dermatology was consulted, and a clinical diagnosis of membranous aplasia cutis congenita (ACC) was made. Skull radiographs were obtained to assess for congenital skull defects. Coronal and sagittal views showed an intact calvarium and no osseous abnormalities (Figures 2, 3). No treatment was required, and the infant has continually met all developmental milestones into his first year of life.

**Figure 1:**
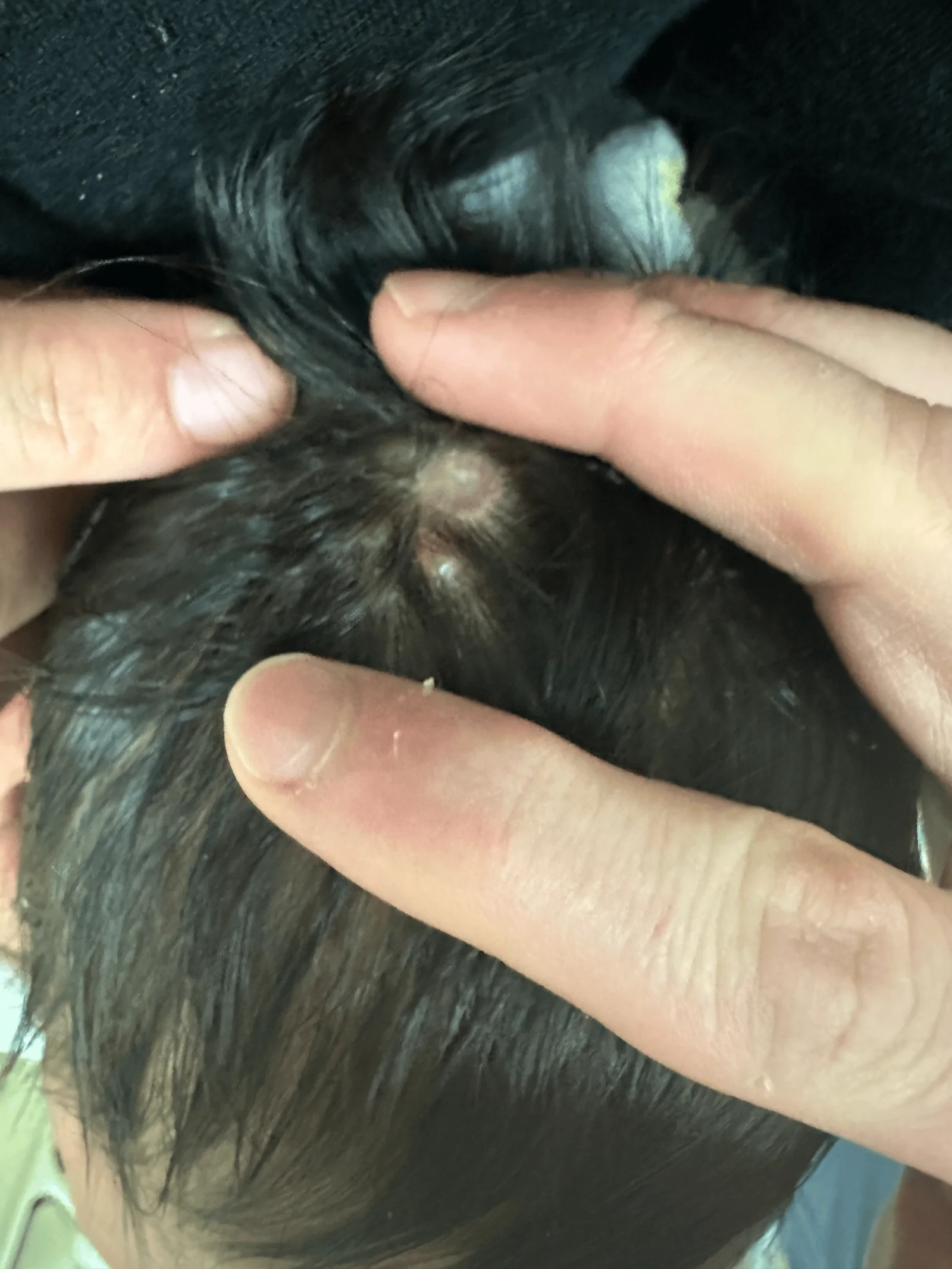
Well-circumscribed, 1-cm, flesh-colored alopecic nodule with a “hair collar sign” or a ring of hypertrichosis surrounding an alopecic lesion, on the right side of the vertex scalp lateral to the midline.

**Figure 2:**
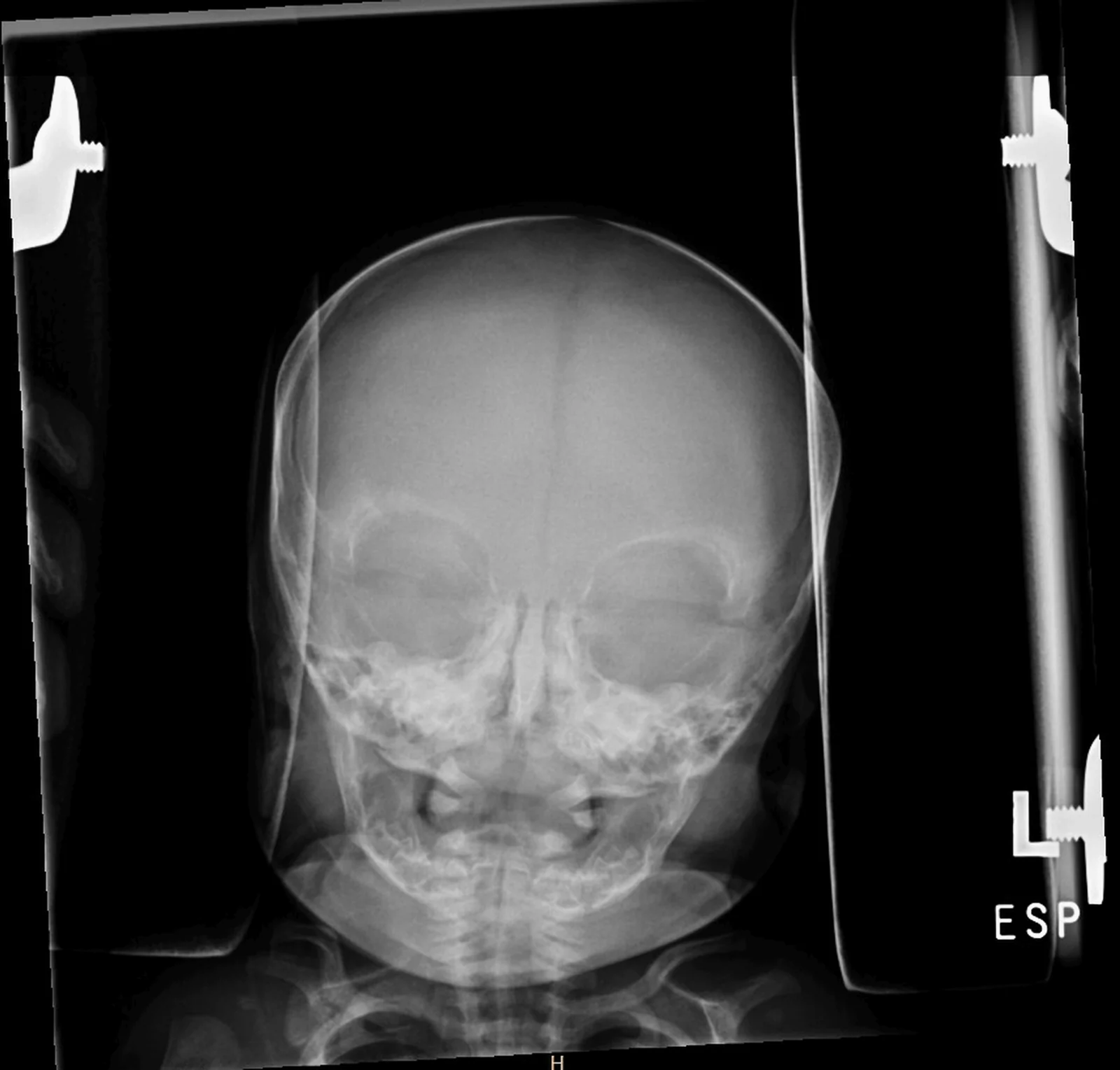
X-ray of the skull, coronal view without abnormality.

**Figure 3:**
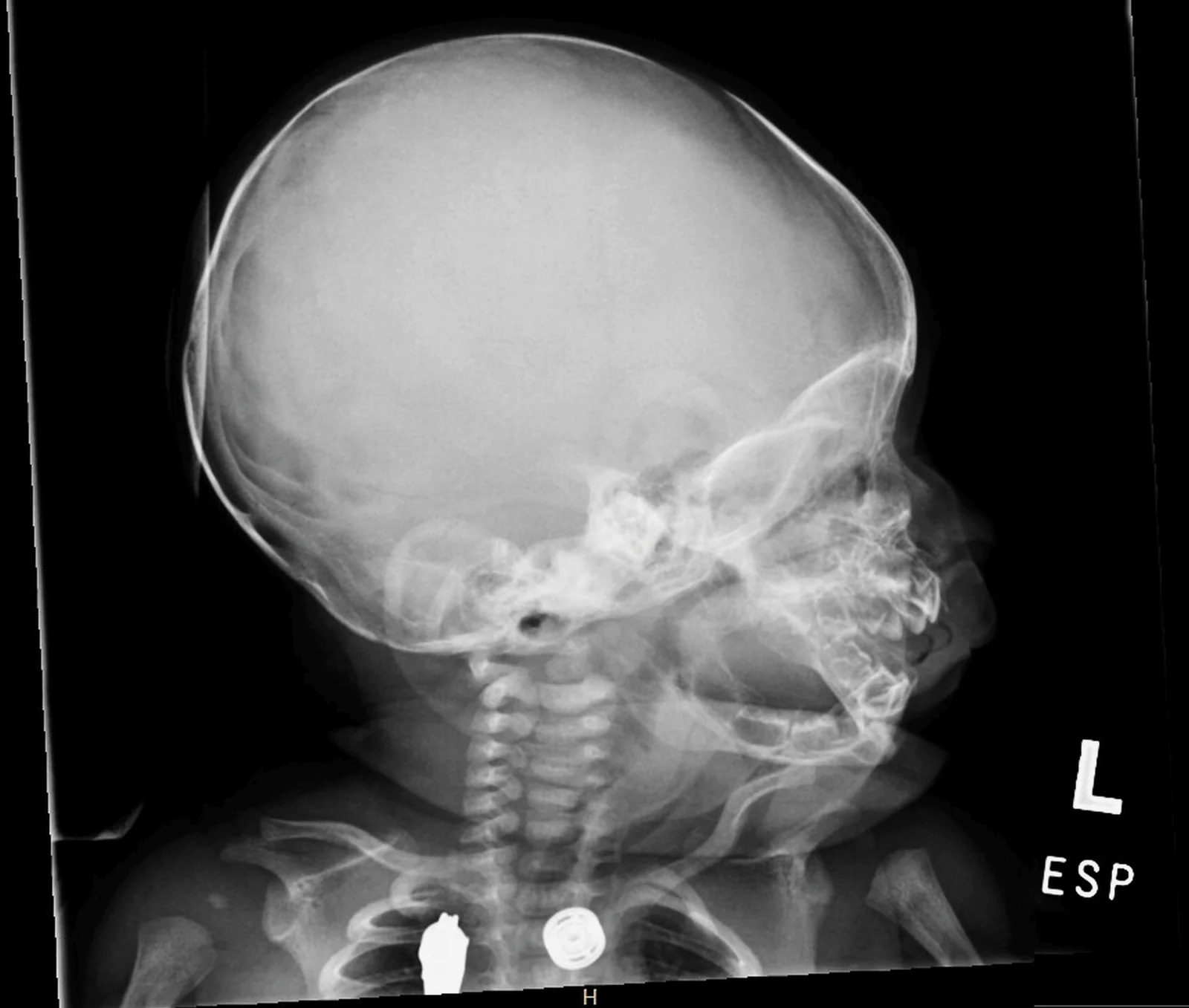
X-ray of the skull, sagittal view without abnormality.

## Discussion

The infant was diagnosed with Frieden group 1 aplasia cutis congenita (ACC), defined as scalp ACC without multiple associated abnormalities, specifically the membranous subtype (Table 1). This clinical diagnosis typically presents as a small (≤1 cm), well-demarcated, round lesion. These lesions are most commonly located between the frontal and parietal regions near the midline, as in this infant (Figure 1) [3,7]. Membranous ACC resembles a blister or cyst that can spontaneously involute, leaving a more indurated papule, as seen in this case. The characteristic “hair collar sign” is strongly associated with this subtype and may indicate underlying osseous abnormalities and therefore warrants further evaluation [3,7].

**Table 1:** Subtypes of ACC as defined by the Frieden classification system. Reproduced from Frieden (1986) with permission from Elsevier [4]. ACC: aplasia cutis congenita

Category	Body area affected	Associated abnormalities	Inheritance
Group 1: scalp ACC without multiple anomalies	Scalp, usually vertex	Cleft lip and palate; tracheoesophageal fistula; double cervix and uterus; patent ductus arteriosus; omphalocele; polycystic kidney; mental retardation; cutis marmorata telangiectatica congenita	Autosomal dominant or sporadic
Group 2: scalp ACC with associated limb abnormalities	Midline scalp	Limb reduction abnormalities; 2-3 syndactyly; clubfoot; nail absence or dystrophy; skin tags on toes; persistent cutis marmorata; encephalocele; woolly hair; hemangioma; heart disease; cryptorchidism; postaxial polydactyly (1 family)	Autosomal dominant
Group 3: scalp ACC with associated epidermal and organoid nevi	Scalp, may be asymmetric	Corneal opacities; scleral dermoids; eyelid colobomas; psychomotor retardation; seizures	Sporadic
Group 4: ACC overlying embryologic malformations	Abdomen, lumbar skin, scalp; any site	Meningomyeloceles; spinal dysraphia; cranial stenosis; congenital midline porencephaly; leptomeningeal angiomatosis; ectopia of ear; omphalocele; gastroschisis	Depends on underlying condition
Group 5: ACC with associated fetus papyraceus or placental infarcts	Multiple, symmetric areas, often stellate or linear, on scalp, chest, flanks, axillae, and extremities	Single umbilical artery; developmental delay; spastic paralysis; nail dystrophy; clubbed hands and feet; amniotic bands	Sporadic
Group 6: ACC associated with epidermolysis bullosa (EB)	-	-	-
Blistering, usually localized, without multiple congenital anomalies	Extremities	Blistering of skin and/or mucous membranes; absent or deformed nails; metatarsus varus; congenital absence of kidney (seen in cases of recessive, dystrophic EB; dominant, dystrophic EB; and EB simplex)	Depends on EB type: may be autosomal dominant or recessive
Widespread skin fragility with congenital anomalies	Large areas on extremities and torso	Pyloric or duodenal atresia; abnormal ears and nose; ureteral stenosis; renal abnormalities; arthrogryposis; amniotic bands; nail dystrophy	Autosomal recessive
Group 7: ACC localized to extremities without blistering	Pretibial areas; dorsal aspects of hands and feet; extensor areas of wrists	None	Autosomal dominant or recessive
Group 8: ACC caused by specific teratogens	Scalp (with methimazole); any area (with varicella and herpes simplex infections)	Imperforate anus (methimazole); signs of intrauterine infection with varicella and herpes simplex infections	Not inherited
Group 9: ACC associated with malformation syndromes	Scalp; any location	Trisomy 13; 4p- syndrome; many ectodermal dysplasias; Johanson-Blizzard syndrome; focal dermal hypoplasia; amniotic band disruption complex; XY gonadal dysgenesis	Varies, depending on specific syndrome

In this patient, the neurologic examination was normal, with symmetric limb movements and normal tone. Skull radiographs obtained to assess for congenital skull defects had no abnormalities (Figures 2, 3). Additional risk factors associated with skull abnormalities include a midline vertex location, lesion diameter greater than 5 cm, or a capillary stain suggestive of an underlying capillary malformation [3]. The patient had no additional risk factors or clinical indicators of an underlying abnormality, so advanced imaging such as CT or MRI was deferred. Most lesions require no intervention and typically heal over several weeks to months. Parents should be counseled that the lesion may remain alopecic throughout the patient’s lifetime.

For membranous ACC, the differential diagnosis is important to consider given that they are often first discovered at birth and some can closely mimic perinatal causes. A keloid scar would present as a shiny, hairless, raised nodule, and a history of fetal scalp electrode (FSE) placement could have been the precipitating factor. However, keloids are often pink or violaceous, with irregular borders, and typically lack the “hair collar” sign [13]. These scars can occur anywhere but are commonly found on the earlobes, shoulders, cheeks, or central chest secondary to high tissue density, increased elasticity, and greater sebaceous gland concentration [13,14]. Asian or African individuals have an increased predisposition to developing keloids due to increased genetic susceptibility [15].

Neonatal herpes simplex virus (NHSV) should also be considered given the maternal history and potential disease severity. NHSV usually presents between two and four weeks of life, though it may occur as late as six weeks [16]. However, affected infants usually present with systemic findings such as fevers, lethargy, and poor weight gain; skin lesions may be absent. When present, NHSV lesions are vesicular with an erythematous base and can cluster at trauma sites such as fetal scalp electrode placements. Approximately 85% of cases are attributed to intrapartum transmission, and many mothers are unaware of their infection; transmission can occur even in the absence of active genital lesions [17,18]. In this case, the mother was receiving appropriate valacyclovir prophylaxis.

Localized epidermolysis bullosa simplex may be present in infancy and is characterized by blistering, most commonly of the palms and soles, although other friction-prone areas might also be affected. Lesions often have erosions with loss of the epidermal layer and may develop crusting [19]. Frieden group 6 ACC is associated with localized epidermolysis bullosa, which presents with lesions on the lower extremities or large areas of the trunk and scalp, with extensive blistering of the skin and mucous membranes [3,4].

## Conclusions

Aplasia cutis congenita (ACC) represents a highly diverse range of neonatal cutaneous defects. While membranous Frieden group 1 ACC often results from spontaneous mutation and is self-limiting, other subtypes may be associated with genetic syndromes and multisystem malformations. Membranous ACC is an important clinical finding to be familiar with and recognize early to prompt evaluation for potential underlying osseous and intracranial abnormalities, particularly when high-risk features are present. Accurate clinical identification is essential to guide management and avoid diagnostic confusion with perinatal trauma such as fetal scalp electrode placement or infectious etiologies, including neonatal herpes simplex virus, as considered in this case.
